# Tolerance and Efficacy of Targeted Therapies After Immunotherapy for Advanced Non-Small Cell Lung Cancers Harboring Oncogenic Alterations: The GFPC-TOXIMAD Study

**DOI:** 10.3390/curroncol33090510

**Published:** 2026-08-27

**Authors:** Thomas Pierret, Jean-Bernard Auliac, Charles Ricordel, Catherine Daniel, Jessica Nguyen, Florian Guisier, Aurélie Swalduz, Hubert Curcio, Anne Laure Desage, Laurence Bigay-Game, Eric Huchot, Lionel Falchero, Hélène Doubre, Olivier Bylicki, Christos Chouaïd, Laurent Greillier

**Affiliations:** 1 Departement of Pneumology, Louis Pradel Hospital, Hospices Civils de Lyon, 69500 Lyon, France; thomas.pierret@chu-lyon.fr; 2 Department of Pneumology, Centre Hospitalier Intercommunal de Créteil, 94010 Créteil, France; jean-bernard.auliac@chicreteil.fr; 3 Department of Pneumology, CHU Rennes, Univ Rennes 1, INSERM, OSS (Oncogenesis Stress Signaling), UMRS 1242, CLCC Eugene-Marquis, 35200 Rennes, France; 4 Thoracic Oncology Service, Thorax Institute Curie Montsouris, Institut Curie, 75005 Paris, France; 5 Departement of Pneumology, CHU BREST, 29200 Brest, France; 6 EA4108 LITIS Lab, QuantIF Team and Inserm CIC-CRB 1404, Service de Pneumologie, Oncologie Thoracique et Soins Intensifs Respiratoires, CHU Rouen, Normandie Université, UNIROUEN, 76031 Rouen, France; 7 Department of Pneumology, Comprehensive Cancer Centre Léon-Bérard, 28, Rue Laennec, 69008 Lyon, France; 8 Department of Medical Oncology, Comprehensive Cancer Center François-Baclesse, 3, Avenue du Général Harris, 14076 Caen, France; 9 Department of Pneumology, CHU Saint-Etienne, 42100 Saint-Etienne, France; 10 Department of Pneumology & Thoracic Oncology, CHU Toulouse, Hôpital Larrey, 31400 Toulouse, France; 11 Departement of Pneumology, CH La Reunion, 97400 Saint Pierre, France; 12 Department of Pneumology, Hôpital Nord-Ouest, 69653 Villefranche-sur-Saône, France; lfalchero@hno.fr; 13 Thoracic Oncology, Hopital Foch, 40, Rue Worth, 92150 Suresnes, France; 14 Department of Pneumology, Hôpital d’Instruction des Armées Saint-Anne, Boulevard Sainte-Anne, 83100 Toulon, France; 15 Multidisciplinary Oncology and Therapeutic Innovations, APHM, INSERM, CNRS, CRCM, Hôpital Nord, Aix–Marseille Université, 13015 Marseille, France

**Keywords:** non-small cell lung cancer, immunotherapy, targeted therapy, adverse events

## Abstract

Background: The sequential use of anti-programmed cell death (ligand)-1 [PD-(L)1] immune-checkpoint inhibitors (ICIs) followed by targeted tyrosine-kinase inhibitors (TKIs) for advanced non-small cell lung cancer (NSCLC) with oncogenic alterations raises questions about tolerance and efficacy. Recent study results suggested an increased risk of adverse events (AEs) with this sequence. Methods: Multicenter retrospective study on advanced NSCLC patients treated with ICIs followed by targeted therapies between 2015 and 2021. The primary endpoint was the rate of grade 3–5 adverse events (AEs). Main secondary endpoints were progression-free survival (PFS), time-to-treatment failure and overall survival (OS). Results: The analysis included 109 patients (most with *EGFR*, 30.3%; *BRAF*, 17.4%; *MET exon-14*, 17.4%; *ALK*, 10.1%; and *RET*, 6.4% gene alterations); 28/109, which is 25.7% of the patients, experienced grade 3/4 AEs and 4/109 (3.7%) experienced grade 5 AEs, leading to the definitive cessation of targeted therapy treatment in 14/32 (44%) of cases; higher grade 3–5 rates were observed with the dabrafenib–trametinib combination (12/16, 75%), capmatinib (4/8, 50%) and crizotinib (8/16, 50%). A last ICI-administration-to-targeted therapy-start interval of <90 days appeared to be associated with grade ≥ 3 AEs (32/82, 39% vs. 0/27 *p* = 0.001). In these sequential strategies, effectiveness of targeted therapies, in this second-line or later setting, appears to be lower than that in the published historical data. Conclusion: According to this analysis, sequential ICI–targeted therapy use for advanced NSCLC appeared to be associated with more grade 3–5 AEs.

## Simple Summary

In patients with advanced lung cancer harboring targetable gene mutations, sequential immunotherapy-targeted therapy was associated with higher rate of adverse events. A short delay between last immunotherapy administration and targeted therapy administration may increase the risk of serious adverse events. This increased toxicity appears to compromise the effectiveness of the targeted treatment.

## 1. Introduction

The treatment paradigm for non-small cell lung cancer (NSCLC) is rapidly changing. Immune checkpoint inhibitors (ICIs) significantly improve survival outcomes of patients with advanced NSCLCs and represent a standard of care [1]. ICIs also confer significant benefit as treatment consolidation after radio-chemotherapy for locally advanced NSCLC [2]. Based on the increasing characterization of molecular aberrations and oncogenic drivers in NSCLCs, it is expected that up to half of patients with advanced NSCLCs will receive oral small-targeted therapies during their successive treatments [3]. Small-targeted therapies are mainly represented by tyrosine-kinase inhibitors (TKIs) that inhibit the activity of tyrosine kinase-dependent oncogenic proteins leading to cell death and clinical activity.

Although TKI toxicity profiles are well-established, little is known about their potential toxicities when administered after ICIs [4,5,6,7,8,9]. Concern is growing that sequential use of ICIs and epidermal growth-factor receptor (EGFR)-TKIs, despite ostensibly unrelated mechanisms of action, may be associated with increased toxicity [10,11,12,13,14]. A database analysis of patients who received nivolumab and any EGFR-TKI(s) during their treatment showed they had an enhanced risk of pneumonitis [11]. Severe hepatitis was also reported in Kirsten rat sarcoma viral oncogene *KRAS^G12C^*-mutated NSCLC patients who received sequential ICI and sotorasib [15,16,17,18]. Among 21 patients who had received this sequence, 76% and 24%, respectively, developed grade ≥ 2 hepatotoxicity and diarrhea. Among the 16 patients with grade ≥ 2 hepatotoxicity, 12 (75%) definitively discontinued sotorasib, nine (56%) of them after dose reductions and rechallenge, and five (32%) received corticosteroids. A larger study analyzed 102 patients who received sotorasib: 48 (47%) sequentially and 54 (53%) in a control group comprising patients who had not received ICIs before sotorasib or had been prescribed chemotherapy between the two [15]. Severe sotorasib-related AEs were significantly more frequent in the sequentially treated group than controls (respectively, 50% versus 13%, *p* < 0.001), including severe hepatotoxicity (33% versus 11%, *p* < 0.006). Severe sotorasib-related AEs typically occurred in patients who received the last ICI infusion within the 30 days preceding sotorasib initiation.

The importance of the interval between the last ICI administration and the start of sotorasib administration was also confirmed in a prospective study [18], showing an increased risk of severe hepatotoxicity, especially in patients with short between-treatment intervals. In that study, serum sotorasib and ICI dosages were available. Sotorasib concentrations did not differ significantly between patients with severe hepatotoxicity or without, but ICI concentrations were higher in those experiencing severe hepatotoxicity. A higher hepatotoxicity rate was also observed with sequential ICIs and crizotinib, compared with crizotinib alone [19], and with sequential ICIs and capmatinib use [20]. Finally, in an analysis exploring risk for selpercatinib-related hypersensitivity in rearranged-during-transfection translocation (*RET*) fusion-positive NSCLC patients, hypersensitivity occurred predominantly in patients previously ICI-treated [21].

Those data raised the hypothesis that small-targeted therapies might trigger severe ICI-mediated toxicities, further suggesting the need to optimize sequencing of targeted therapies and ICIs.

The primary objective of this study was to evaluate severe AEs occurring after sequential ICI–TKIs, in the context of advanced NSCLCs with oncogenic drivers. The secondary objectives were to describe the type and severity of these AEs, their impact in terms of temporary or permanent treatment discontinuation, and treatment efficacy.

## 2. Methods

This retrospective multicenter analysis used the Groupe Français de Pneumo-Cancerologie (GFPC) database to identify patients whose advanced NSCLCs harbored oncogenic addiction and were treated with ICIs (pembrolizumab, nivolumab, atezolizumab or durvalumab) followed by TKIs between 2015 and 2021. The GFPC database is a real-life nationwide network of approximately 40 centers across France that stores the treatment-efficacy and -tolerance data of treated NSCLC patients. For this analysis, the main inclusion criteria were: age > 18 years, advanced NSCLC and *EGFR*, v-raf murine sarcoma viral oncogene homolog B1 (*BRAF*), human epidermal growth-factor receptor-2 (*HER2*), mesenchymal-to-epithelial transition (*MET*) exon-*14* jump mutations, or rearranged-during-transfection translocation (*RET*), neurotrophic receptor tyrosine kinase (*NTRK*), proto-oncogene tyrosine-protein kinase-1 (*ROS1*) or anaplastic lymphoma kinase (*ALK*) rearrangement. Patients who received concomitant ICIs and targeted therapy were excluded, as they were mostly treated within the framework of clinical trials.

The information collected included: patient characteristics; comorbidities; NSCLC characteristics; treatment received prior to TKI; last-ICI-administration-to-targeted therapy-start interval; last-ICI-administration-to-AE-appearance interval on targeted therapy; AE type, grade and impact on treatment; response rate; progression-free survival (PFS); and overall survival (OS). Analysis focused on groups having at least five patients and the TKIs still being used. Data were updated until patient death or data lock on 15 September 2024.

The primary endpoint was the rate of grade 3–5 toxicities, according to the Common Terminology Criteria for Adverse Events [22].

Categorical parameters were compared using Fisher’s exact test (for an expected event frequency of <5) or Chi-square test. Continuous variables were compared with Student’s *t*-test, Mann–Whitney U-test or Kruskal–Wallis test. Odds ratios (ORs) were calculated, and Haldane correction was applied where appropriate. Univariate and multivariate logistic-regression analysis identified variables associated with severe AEs. Median (range) follow-up duration with corresponding 95% confidence intervals (CIs) were calculated by reverse Kaplan–Meier methodology.

The study was approved by the Institutional Review Board of the French Society for Respiratory Medicine (CEPRO 2023-047 on 7 December 2023) and was conducted in accordance with good clinical practice guidelines and the Declaration of Helsinki’s ethics principles for medical research. In accordance with French law, all patients received oral information about data collection.

## 3. Results

A total of 109 patients were included: median age 67 (range 39–91) years, 54.1% female, 44% former smokers and 41% never-smokers. Histology was predominantly adenocarcinoma (92.7%); 41.3% with anti-programmed cell death (ligand)-1 [PD-(L)1] status ≥ 50% (Table 1). At diagnosis, only 66% had molecular testing results; at study inclusion, they were available for all the patients, based on tissue (83%), liquid biopsy (12%) or both (4%). The most common mutations were *EGFR* (28.4%), *BRAF* (17.4%), *MET* exon-14 (17.4%), *HER2* (16.5%), *ALK* (10.1%), and *RET* (6.4%).

Main toxicities occurring during ICI (with or without chemotherapy) administration are reported in Table 2.

At the start of targeted therapy, they had received a median of 1 (range 1–7) previous systemic therapy line(s). At targeted therapy initiation, 65.1% of patients had an Eastern Cooperative Oncology Group performance status (ECOG-PS) of 0/1; 22.9% were taking corticosteroids, with median dose at 60 mg/day. The most common TKIs were osimertinib (*n* = 25), crizotinib (*n* = 16), dabrafenib + trametinib (*n* = 16), lorlatinib (*n* = 5) and pralsetinib (*n* = 5) (Table S1)**.**

The median last ICI-administration-to-targeted therapy onset interval was 39 (range 1–1187) days, with 43/109 (39.4%) and 86/109 (78.9%) patients having intervals, respectively, of <30 and <90 days.

During targeted therapy, 28/109 (25.7%) of the patients experienced grade 3/4 AEs and 4/109 (3.7%) grade 5, leading to the definitive cessation of targeted therapy treatment in 14/32 (44%) of cases (Table 3). The deaths were related to respiratory (*n* = 2), digestive (*n* = 1), and cardiovascular (*n* = 1) events. Digestive and hepatobiliary AEs occurred after a median of 29 and 35.5 days, respectively; cardiovascular and neurological AEs occurred later, after a median of 97.5 and 111 days, respectively. No toxicity was observed in patients treated with first- and second-generation anti-EGFR-TKIs and antibody conjugates, such as amivantamab, trastuzumab alone or trastuzumab–deruxtecan; there was, especially, no evidence of interstitial pneumonia. The TKIs most likely to cause toxicity after ICIs appear to be capmatinib 4/8 (50%), crizotinib (8/16, 50%) and dabrafenib–trametinib, 12/16 (75%).

For efficacy, the analysis was restricted to groups with more than five patients. In this setting, targeted therapy efficacy seemed to be lower than that in the published data (Table 4). Analyzing the impact of the time between the last ICI administration and target therapy onset, an interval of <90 days appeared to be associated with more grade ≥ 3 AEs; with 32/82, that is, 39% of patients having an interval of less than 90 days versus none with an interval of > 90 days (*p* = 0.001).

Univariate analysis failed to identify any other factor linked with grade ≥ 3 AE occurrence.

## 4. Discussion

This retrospective analysis of 109 patients with advanced NSCLCs harboring oncogenic alterations who had received targeted therapy after ICIs revealed that 25.7% and 3.7% of the patients experienced grade 3/4 and grade 5 AEs, respectively, leading to the definitive cessation of targeted therapy treatment in 14/32 (44%) of cases. Crizotinib and combination dabrafenib–trametinib had the higher grade 3–5 AE rates. A short interval between the last ICI administration and targeted therapy appeared to be associated with more grade ≥ 3 AEs, and in this setting, the efficacy of targeted therapy appears lower compared to published data.

Previously reported results had already underlined the enhanced AE rate on TTs used sequentially with ICIs. A meta-analysis [5] showed that the grade 3–5 toxicity rate was 57%, especially in more severe hepatic toxicities. Another large retrospective analysis of 126 advanced, *EGFR*-mutated NSCLCs showed that 15% (6/41) of the patients treated with ICIs followed by osimertinib experienced severe AEs as opposed to none of the 29 patients treated with osimertinib followed by ICIs, while the two groups had no evident clinical differences. Those AEs occurred within the first few weeks after starting osimertinib, regardless of ICI duration, with some patients having been given only one ICI dose. The median interval between the last ICI dose and osimertinib onset was 23 days [6].

A short interval between the two therapeutic sequences seems, as herein, to increase toxicity frequency [4]. That observation was also shown for crizotinib, with a median interval of 30 days for grade 3/4 aspartate aminotransferase/alanine aminotransferase elevation, compared to 50 days for patients without elevated transaminases [19]. Similar results were obtained with sotorasib: grade ≥ 3 AEs were more frequent (28%) in patients who had received their last anti-PD-(L)1 ICI infusion within the 12 weeks preceding sotorasib initiation and reached 58% when the interval was <30 days [15,16,17,18,19]. Finally, 82.4% (14/17) of hypersensitivity reactions to selpercatinib began within 3 months after the last ICI infusion [21].

In the absence of a control cohort, it appears difficult to determine the proportion of AEs related to the ICI-TKI sequence. In second-line pivotal trials, the proportion of patients with grade 3 or higher AEs was 23% with osimertinib [23] and 56% with the dabrafenib-trametinib combination [24]. These rates are 20% and 75%, respectively in our study.

Similarly, the efficacy reported in our study must be interpreted in the context of heavily treated patients who have already received at least one line of therapy.

The mechanisms explaining the synergistic toxicities of these ostensibly distinct therapies are not clear and warrant future investigation. Recently, EGFR-TKIs were shown to differentially enhance T-cell-mediated killing of tumor cells by increasing both basal and interferon-gamma induced major histocompatibility complex class-I presentation [13]. A combination of factors, including off-target effects on T-cell activation, alterations in the immune microenvironment, and differences in TKI tissue distribution, may contribute [25]. For example, ALK TKIs promote immunologic remodeling with increased T-cell tumor infiltration; activation of tumor-specific T cells followed by increased infiltration upon TKI initiation represents one potential mechanism for the enhanced toxicity [26]. Thus, it is possible that osimertinib has underappreciated immunomodulatory effects.

Another pertinent question arising through these overlapping toxicities is whether these enhanced toxicities are a drug or class-effect interaction with ICIs. More severe AEs following ICIs have been reported for TKIs (i.e., osimertinib, selpercatinib and crizotinib) and *KRAS^G12C^* inhibitor (i.e., sotorasib). Concerning sequential administrations, several observations indicated the specific risk of osimertinib, compared with first- (i.e., erlotinib or gefitinib) or second-generation (i.e., afatinib) EGFR-TKIs [27,28].

Those findings support the benefit for all patients of comprehensive molecular testing at initial diagnosis. The National Comprehensive Cancer Network guidelines recommended *ALK*, *ROS1*, *EGFR* and *BRAF* testing for all patients at diagnosis [29]. Despite that recommendation, patients are often under-genotyped or even not genotyped at diagnosis, as illustrated by the recent multinational retrospective study [30]. The molecular-testing rate for 1440 patients from eight countries varied widely, with 54% to 91% of patients having undergone at least one molecular test and 9% to 46% of patients with non-squamous NSCLCs without any molecular testing. That observation is concerning, given the known OS improvement for patients who receive TKIs chosen based on their molecular profiles [26]. The lack of comprehensive testing is further compounded by the fact that, even when patients are tested and have a positive predictive marker, they are often not given the indicated targeted therapy [31,32].

Our analysis has several limitations. The conclusions are limited by the retrospective study design, AE determination by the treating clinician, no central monitoring, and the modest number of patients limiting our ability to draw definitive conclusions.

Furthermore, there is no control group, which makes it impossible to assess the proportion of adverse events related to the ICI-TKI sequence, and the cohort is already somewhat old, while progress has been made in the management of these adverse events. Further investigations on toxicity mechanisms and the relevant clinical circumstances are needed to better guide patient care, particularly the contribution of prophylactic corticosteroid use or inserting chemotherapy between ICIs and targeted therapy.

In conclusion, according to the results of this analysis, sequential ICI-then-targeted therapy administration to treat advanced NSCLCs harboring oncogene alterations appears to be associated with a higher risk of severe AEs, especially when the ICI-to-targeted therapy interval was <90 days.

## Figures and Tables

**Table 1:** Characteristics.

Characteristic	*n* = 109 (100%)
Age (median, range) years	67 (39–91)
Sex	
Male	50 (45.9%)
Female	59 (54.1%)
Smoking history	
Active smoker	14 (12.8%)
Former smoker	48 (44.0%)
Never-smoker	45 (41.3%)
Unknown	2 (1.8%)
Histology	
Adenocarcinoma	101 (92.7%)
Other	8 (7.3%)
PD-L1 status	
<1%	20 (18.3%)
1–49%	17 (15.6%)
>50%	45 (41.3%)
Not done	27 (24.8%)
Mutation	
*EGFR*	33 (30.3%)
*ALK*	11 (10.1%)
*ROS1*	1 (0.9%)
*BRAF*	19 (17.4%)
*RET*	7 (6.4%)
*MET exon-14*	19 (17.4%)
*HER2*	18 (16.5%)
Others	1 (1%)

Values are *n* (%) unless stated otherwise.

**Table 2:** Main toxicities on immune checkpoint inhibitors (ICIs) (± Chemotherapy), laboratory findings at targeted therapy onset.

Toxicity on ICI (>5%)	*n* = 109
Anemia	31 (28.4%)
Lymphopenia	21 (19.3%)
Cutaneous	16 (14.7%)
Renal insufficiency	13 (11.9%)
Thyroid dysfunction	10 (9.2%)
Hepatic cytolysis	15 (13.8%)
Hypothyroidism	7 (6.4%)

Values are *n* (%) unless stated otherwise.

**Table 3:** Severe adverse events according to target therapy.

Targeted Therapy	*n*	Grade 3–4	Grade 5	Stopped Treatment
Osimertinib	25	4 (16%)	1 (4%)	1 (4%)
Dabrafenib–trametinib	16	10 (62.5%)	2 (12.5%)	3 (18.8%)
Crizotinib	16	7 (43.8%)	1 (6.2%)	5 (31.3%)
Pralsetinib or selpercatinib	8	1 (12.5%)		1 (12.5%)
Capmatinib	8	4 (50%)		2 (25%)
Others	42	2 (4.8%)		2 (4.8%)

Values are *n* (%) unless stated otherwise. Only the tolerance of target therapies received by more than five patients are reported.

**Table 4:** Efficacy according to targeted therapy received.

Targeted Therapy	ORR	DCR	PFS	OS
			Months	
Osimertinib (EGFR)	(4/25) 16%	44%	2.1	7.3
Dabrafenib–trametinib (BRAF V600E)	(12/16) 75%	87.5%	9	19
Crizotinib (MET exon 14)	(4/11) 36.4%	73%	5.7	14.7
Capmatinib (MET exon 14)	(6/8) 75%	100%	7.7	14
Pralsetinib/selpercatinib Vandetanib (RET)	(5/7) 71.4%	86%	13.1	26
Lorlatinib (ALK)	(1/5) 20%	80%	5	9.7

ORR, objective response rate; DCR, disease-control rate; PFS, progression-free survival; OS, overall survival. Values are *N* (%) unless stated otherwise.

## Data Availability

The data presented in this study are available on request from the corresponding author.

## Supplementary Materials

The following supporting information can be downloaded at: https://www.mdpi.com/article/10.3390/curroncol33090510/s1, Table S1: Targeted therapy prescribed.
