# Supplementary material for: Tolerance and Efficacy of Targeted Therapies After Immunotherapy for Advanced Non-Small Cell Lung Cancers Harboring Oncogenic Alterations: The GFPC-TOXIMAD Study
Source: Curr Oncol. 2026 Aug 27;33(9):510. doi: 10.3390/curroncol33090510 (PMC13605073; doi:10.3390/curroncol33090510)
Supplement: Supplementary file 1 [file curroncol-33-00510-s001.zip › curroncol-4426266-supplementary.pdf]

Table S1. Targeted therapy prescribed.

	n = 109
EGFR	**33 (30.3%)**
Osimertinib	25 (22.9%)
Gefitinib or erlotinib	5 (4.9%)
Afatinib	1 (0.9%)
Amivantamab	2(1.6%)
MET exon-14	**19 (17.4%)**
Crizotinib	11 (10.1%)
Capmatinib	8 (7.3%)
BRAF	**19 (17.4%)**
Dabrafenib–trametinib	16 (14.7%)
Dabrafenib	2 (1..8%)
HER2	**18 (16.5%)**
Trastuzumab	7 (6.5%)
Trastuzumab–deruxtecan	6 (5.5%)
Trastuzumab–pertuzumab	1 (0.9%)
Pyrotinib	2 (1.8%)
ALK	**11 (10.1%)**
Lorlatinib	5 (4.6%)
Crizotinib	5 (4.%)
Ceritinib	1 (0.9%)
RET	**7 (6.4%)**
Pralsetinib	5 (4.9%)
Selpercatinib	1 (0.8%)
Vandetanib	1 (0.8%)
***ROS1*, lorlatinib**	**1 (0.9%)**
***NTRK*, larotrectinib**	**1 (0.9%)**

Oncogenes: *EGFR*, epidermal growth-factor receptor; *MET*, mesenchymal-to-epithelial transition; *BRAF*, v-raf murine sarcoma viral oncogene homolog B1; *HER2*, human epidermal growth-factor receptor-2; *ALK*, anaplastic lymphoma kinase; *RET*, rearranged-during-transfection translocation; *KRAS*, Kirsten rat sarcoma viral oncogene; *ROS1*, proto-oncogene tyrosine-protein kinase-1; *NTRK*, neurotrophic receptor tyrosine kinase
Values are *N* (%) unless stated otherwise.
